# Scale of Adherence to Good Hospital Practices for COVID-19: Psychometric Properties

**DOI:** 10.3390/ijerph191912025

**Published:** 2022-09-23

**Authors:** Silmara Meneguin, Camila Fernandes Pollo, Ediana Preisler Melchiades, Melissa Santiloni Montanha Ramos, José Fausto de Morais, Cesar de Oliveira

**Affiliations:** 1 Department of Nursing, Botucatu Medical School, Paulista State University, Botucatu 18618-687, SP, Brazil; 2 Faculty of Mathematics, Federal University of Uberlândia, Uberlândia 38400-902, MG, Brazil; 3 Department of Epidemiology & Public Health, University College London, London WC1E 6BT, UK

**Keywords:** COVID-19, methodological study, psychometrics, validation study

## Abstract

To avoid hospital transmission, all COVID-19 prevention measures should be followed. This study aimed to evaluate the psychometric properties of a novel scale developed to assess adherence to good practices for COVID-19 in the hospital setting. A methodological cross-sectional study was conducted at a public hospital in the state of São Paulo, Brazil, with 307 healthcare providers. Data were collected using a questionnaire addressing sociodemographic/occupational data and the Adherence to Standard Precautions for COVID-19 scale. Cronbach’s alpha coefficients and the intraclass correlation coefficients were used to measure internal consistency and temporal stability (test-retest analysis), respectively. Concurrent validity was evaluated using Spearman’s correlation coefficients between the scores of the overall scale and its domains. Factorial structure was evaluated using exploratory factor analysis and goodness-of-fit of the model was tested using confirmatory factor analysis. Cronbach’s alpha coefficients for the scale and its domains were higher than 0.7, except the psychosocial domain (0.61). All intraclass correlation coefficients were higher than 0.7. Strong correlations were found between the total score and the personal (0.84) and organizational (0.90) domains of the scale and a good correlation was found with the psychosocial domain (0.66). The fit of the multidimensional model was satisfactory for all parameters and the three-dimensional structure of the scale was confirmed by the fit of the factor loadings. The novel scale is a valid and reliable instrument for assessing adherence to good hospital practices for COVID-19.

## 1. Background

In March 2020, the World Health Organization declared COVID-19 a global pandemic, placing all countries in a state of maximum alert due to the exponential growth in the number of cases and rapid dissemination of the disease [1].

COVID-19 exerted impacts on the individual, interpersonal, organizational and extra-organizational levels, with considerable concerns for healthcare providers regarding infection, mortality, the need for social distancing and the socioeconomic crisis resulting from the vast spread of the virus [2]. As a consequence, an unprecedented global health crisis took place [3,4].

The high mortality and infection rates in combination with difficulties in containing the spread of the virus contributed to an increased level of awareness in the general population with regards to the health risks. 

A closer proximity to the COVID-19 pandemic, for health professionals, may have increased the negative emotions associated with an increased risk scenario considerably [5]. Therefore, the World Health Organization has emphasized the importance of adopting protective measures to avoid infection and dissemination, as healthcare providers are the main vehicles for the transmission of microorganisms due to direct and indirect contact with infected patients [6].

The adequate use of standard precautions is essential for healthcare services that manage patients with COVID-19. The spread of infections at health services can be avoided through adherence to good standard practices. The lack of adherence to such practices by healthcare workers, especially in a pandemic scenario, is likely to result in an increased risk of infectious disease [7]. Such precautions involve hand sanitizing and the use of personal protective equipment [8]. To avoid hospital transmission, all COVID-19 prevention measures should be followed. 

Healthcare workers, in particular nurses, are more exposed to infections due to their working environment than the general population [9,10]. Adherence to standard precautions at all times, independent of a case being suspected or confirmed, is considered the most effective method to prevent cross-infection [11]. Despite the overwhelming evidence on the efficacy of adherence to standard precautions, adherence to such practices is still insufficient among healthcare workers. Furthermore, adherence levels vary by different standard precaution components [12,13,14].

Healthcare workers were at the frontline in treating COVID-19 patients and helping to control the spread of the virus. However, any lack of adherence to standard precautions could increase the level of infection among such professionals and, ultimately, could lead to a collapse of the health system [15].

The critical condition of patients with a suspicion or confirmation of COVID-19 is generally accompanied by a variety of complications that cause an excessive workload for the entire health team, requiring commitment and adherence to good hospital practices to avoid the dissemination of the virus. 

Adherence is a dynamic, multifactorial process that results from a set of determinants dependent on subjective, organizational and work-related factors. It is a complex decision-making process mediated by psychological traits, cognitive level, beliefs, values and sociocultural context [16].

Therefore, it is essential to assess adherence to standard precautions through the use of valid, reliable instruments for use when providing care for patients with COVID-19 [17,18,19]. Assessment tools are fundamental to the recognition of gaps in knowledge on the part of healthcare providers. However, the literature offers few instruments for assessing adherence to precautions for COVID-19. Most adherence assessment tools were designed for other diseases and do not address specificities inherent to the transmission of COVID-19 [20,21,22,23,24,25,26,27,28,29,30,31].

This lack of validated tools for the assessment of adherence to good hospital practices for COVID-19 motivated the development of the present investigation. The development of the items and dimensions of the Adherence to Good Hospital Practices for COVID-19 scale (AGHPC) was based on a thorough review of the existent literature followed by a pilot conducted with sixteen nursing staff. The scale with 51 items was then evaluated by seven experts and the items that showed content validity of ≥0.83 were kept. In the semantic analysis, carried out with 35 health professionals, there were no changes requested and the comprehensiveness level was 0.87%. The AGHPC scale with 47 items and three dimensions (personal, organizational and psychosocial) developed in the present study achieved satisfactory content and face validity, meeting the parameters established in the literature [32].

Therefore, the aim of this study was to evaluate the psychometric properties of a novel scale designed to assess adherence to good practices for COVID-19 in the hospital setting.

## 2. Methods

### 2.1. Study Design

A methodological, cross-sectional study with a quantitative approach was conducted in the period from April 2020 to October 2020. Data were collected from a public hospital in the state of São Paulo that serves as a reference center for the treatment of patients with a suspicion or confirmation of COVID-19. This public hospital has approximately 490 beds, of which 54 are in the adult, pediatric and neonatal intensive care units.

### 2.2. Study Population

The sample was composed of female and male healthcare workers aged 18 years or older who worked in sectors dedicated to the treatment of patients with a suspicion or confirmation of COVID-19 and who agreed to participate in the study. Participants who did not complete the questionnaire were excluded. 

Although there is no gold standard for sample size when validating a new instrument, the recommendation is that the sample should be at least four to ten times the number of items with a minimum sample of 100 individuals, to ensure an appropriate validity analysis [33,34]. Our final analytical sample was composed of 307 participants.

### 2.3. Data Collection Procedure

A two-part data collection instrument was used. The first part addressed sociodemographic and occupational aspects and the second was the scale for the assessment of adherence to good hospital practices. The scale has 47 items distributed among three domains: personal, organizational and psychosocial. The response options are scored on a five-point scale: never = 1, rarely = 2, sometimes = 3, often = 4 and always = 5. The total ranges from 47 to 235 and is categorized as follows: ≤58 points = minimal adherence; 59 to 117 points = partial adherence; 118 to 175 points = moderate adherence; and 176 to 235 = maximal adherence. The score range categorization was based on the median value and the interquartile interval for the total scale score.

The researchers delivered the data collection instrument and statement of informed consent in a sealed envelope and the material was collected after being filled out. The average response time for the questionnaire was 20 min. 

Thirty-one participants were reassessed between 7 and 14 days after their first interview to evaluate temporal stability (test-retest analysis). 

### 2.4. Statistical Analysis

For the characterization of the sample, continuous variables were expressed as means and standard deviations and categorical variables were expressed as percentages. 

### 2.5. Construct Validity

Exploratory factor analysis was performed to determine the factorial structure of the scale. The adequacy of the sample was calculated using the Kaiser–Meyer–Olkin test and Bartlett’s test of sphericity. These two tests allow one to assess whether it is adequate to conduct a factor analysis or the sampling adequacy. The factorability of the data was determined by a value of KMO > 0.50 and significance of the Bartlett’s test of sphericity (*p* < 0.05) [35].

The factorability was assessed using the Horn parallel analysis method, in which factors with eigenvalues greater than 1.0 were extracted. Indeed, in the exploratory factor analysis, two criteria were considered for the maintenance of questions/factors: absolute factor loading above 0.3 in only one factor and factors with three or more questions.

In the confirmatory factor analysis, the following fit indices were used: the Comparative Fit Index (CFI) and the Tucker–Lewis Index (TLI), with values between 0.90 and 0.95 indicating acceptable fit levels and values ≥ 0.95 indicating a good level of fit of the model; the root mean squared error of approximation (RMSEA) with values ≤ 0.05 or 0.08 indicating a good fit and rejection of the null hypothesis (*p* < 0.05) and the standardized root mean squared residual (SRMR) with values of 0.08 or lower indicating a good fit. The minimum value of the discrepancy function based on the chi-square (CMIN) and CMIN/df was also estimated. In addition, the standardized loading of the items should be greater than 0.3 [36].

### 2.6. Convergent Validity

Tests of convergent validity assume that the measures under study are related to the same concept. It may be that two measures correlate not because the two capture the same concept, but because casual relationships exist that drive the correlation [37].

Spearman’s correlation coefficients were calculated between the scores of the overall scale and its domains. Values < 0.4, 0.4 to 0.6 and >0.6 were considered weak, moderate and strong correlations, respectively [38].

### 2.7. Reliability

Reliability analysis was performed based on internal consistency and temporal stability (test-retest analysis). Internal consistency of the scale and its dimensions was evaluated using Cronbach’s alpha coefficients, with values higher than 0.70 considered acceptable [39]. Temporal stability was analyzed using intraclass correlation coefficients (ICC), with values < 0.4, 0.4 to 0.6, 0.6 to 0.75 and >0.75, respectively, considered weak, fair, good and excellent [40].

Statistical analysis was performed with the aid of SPSS version 25^®^ (SPSS Inc., Chicago, IL, USA). The significance level was set at 5% (*p* < 0.05).

### 2.8. Ethical Issues

This study received approval from the Human Research Ethics Committee of the Botucatu School of Medicine (certificate number: 4.007.407) and was conducted in accordance with norms governing research involving human subjects and it was structured according to guideline recommendations Standards for Quality Improvement Reporting Excellence (SQUIRE 2.0) [41] and COnsensus-based Standards for the selection of health Measurement INstruments (COSMIN) [42].

## 3. Results

The final sample was composed of 307 participants. Most participants were women (84%), living with a partner (70%), working at intensive care (35.5%) as nursing technicians (48.5%), Table 1.

The total score of the Adherence to Good Hospital Practices for COVID-19 scale in the present study was 177 as follows: 50 points at the personal, 96 at the organizational and 31 points at the psychosocial domain. 

### 3.1. Construct Validity 

#### 3.1.1. Exploratory Factor Analysis 

**Figure 1:**
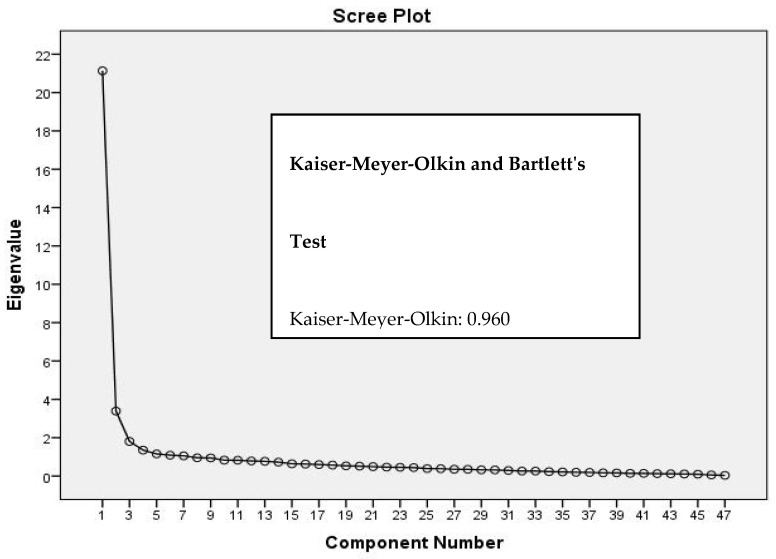
Scree plot of Horn’s parallel analysis.

Exploratory factor analysis revealed three oblique factors with an eigenvalue equal to or greater than 1 (Figure 1), which were confirmed by Horn’s parallel analysis. For the evaluation of dimensionality, exploratory factor analysis was performed to determine how the items were grouped. Horn’s parallel analysis indicated three-dimensionality for the scale. The adequacy of the sample for the analysis was demonstrated by the Kaiser–Meyer–Olkin measure (0.96) and the result of Bartlett’s test of sphericity (10,680.7) (*p* < 0.01). These results demonstrated suitability of the data matrix for factor analysis.

Table 2 displays the data from the exploratory factor analysis using the number of factors identified in the scree plot. The factor loadings were presented within each respective domain. The data show a total variance of 78.2% (51.52% for the organizational domain, 7.5% for the psychosocial domain and 13.28% for the personal domain). All items had values greater than 0.3.

#### 3.1.2. Confirmatory Factor Analysis

The three-dimensionality of the instrument was indicated by the confirmatory factor analysis. The fit indexes results are: RMSEA (0.072; *p* = 0.001), CFI (0.996), TLI (0.995), CMIN (2428.3; *p* ≤ 0.001), CMIN/DF (2.578) and SRMR (0.082). The standardized loadings of the items resulted in >0.3.

Table 3 displays the correlations between the total score of the scale and its domains. Strong correlations were found between the total score and both the personal domain (0.84) and organizational domain (0.90) and good correlation was found with the psychosocial domain (0.66).

Table 4 displays Cronbach’s alpha coefficients for the scale and its respective domains. Correlation values were considered satisfactory (>0.70), except for the psychosocial domain (0.61).

Table 5 shows the median (25th–75th percentile) of the total scale and domain scores on two occasions for the evaluation of temporal stability (test-retest analysis). All ICCs were higher than 0.7, demonstrating satisfactory temporal stability.

The validation of the scale based on the analyses of its psychometric properties confirmed 47 items that represent the construct.

## 4. Discussion

The purpose of this study was to examine the psychometric properties of a novel scale developed to assess adherence to good practices for COVID-19 in the hospital setting with 47 items. The original scale content validation study included the participation of physicians, physical therapists and nursing staff.

It is difficult to measure adherence to good practices in the hospital setting. Therefore, there was a clear need to develop an instrument capable of assessing adherence amidst the COVID-19 pandemic. In the present study, an exploratory factor analysis was conducted to obtain a reasonable and comprehensive model that in turn would produce a good theoretical and conceptual framework with a sound structure [43]. Our exploratory factor analysis has confirmed the tridimensionality of the Adherence to Good Hospital Practices for COVID-19 (AGHPC) scale proposed by the authors. However, the factor 3 showed weaker correlations, lower internal consistency and lower explained variance i.e., 7.5%. After the confirmatory factor analysis, all the quality criteria for model adjustment were considered satisfactory. The final model showed high adjustment quality levels for all tests (chi-squared = 2428.3, CMIN/DF = 2.578, RMSEA = 0.072, SRMR = 0.082, Tucker–Lewis index = 0.995, CFI = 0.996, quality of adjustment index = 0.993 and AGFI = 0.991). In general, it is recommended that the confirmatory factor analysis should be performed with three or more items per factor [44].

Construct validation was also performed using concurrent validity between the overall scale and its domains. Strong correlations were found between the scale and both the organization domain (0.90) and personal domain (0.84) and a good correlation was found with the psychosocial domain (0.66).

As for determining the reliability of the scale, the internal consistency was evaluated using Cronbach’s alpha coefficient, which is an important method for assessing the reliability of instruments with multiple items [45]. Cronbach’s alpha for the overall scale was considered excellent (0.96). A high alpha was found for the organizational domain (0.95), 0.88 was found for the personal domain and the coefficient for the psychosocial domain, which has fewer items, was 0.61. It is important to stress that Cronbach’s alpha coefficients are strongly influenced by the number of items on a measurement instrument [45].

Satisfactory results were found with regards to temporal stability (test-retest), with ICC values higher than 0.70 for the overall scale and its respective domains. Therefore, the adherence scale had good reproducibility. As an important methodological note, people’s moment-by-moment experienced emotions are influenced by transitory situational factors and exhibit very low stability [46]. With regards to sample size, the retest was conducted in accordance with the COSMIN checklist, i.e., less than 30 participants, 30 to 49 participants, 50 to 99 participants and greater than 100 participants denote poor, fair, good and excellent methodological quality of the studies, respectively [47]. On the other hand, a more recent reference for the retest sample size derived from Power Analysis and Sample Size (PASS) software was used [48].

In this study, the adherence rate to the scale was 54.5%. The data were collected during the first months of the pandemic when there was little knowledge about the coronavirus. A study conducted in Italy showed that the adoption of standard precautions is crucial to prevent the spread of infection and protect the health of health workers. However, such an approach will only be effective if it is adhered to by all health professionals [15].

The present study has limitations that should be acknowledged. First, this is a study conducted in only one health center. Second, it has a cross-sectional design which does not allow us to imply causality. Third, the resources available to fight COVID-19 could vary within Brazil and between countries. Fourth, we were not able to perform a responsiveness analysis due to restrictions inherent to the pandemic. Although the present study has an adequate sample size, our findings should be interpreted with caution and should not be generalized to all health care workers. It is also important to highlight the lack of studies on the theme, which made it difficult to compare our results. Finally, the psychometric properties of the measure should be examined in the general population of other countries.

The main contribution of this study was the development of a hospital scale for assessment of the adherence of healthcare providers to good practices to prevent the transmission of COVID-19. This study could also contribute to the development of interventions that seek the establishment of safe care within the hospital, consequently minimizing the occurrence of morbidity and mortality.

## 5. Conclusions

The Scale of Adherence to Good Hospital Practices for COVID-19 is a valid, reliable instrument for assessing adherence to good hospital practices for COVID-19. This novel scale offers the opportunity to collect data that enable the design and implementation of interventions that improve the quality of hospital care for patients with COVID-19.

Although the results were statistically significant, similar studies should be conducted to confirm the generalizability of the results, including in other countries.

## Figures and Tables

**Table 1:** Sociodemographic characteristics of participants (*n* = 307).

Variable	*n* (%)
Sex	
Female	258 (84.0)
Male	49 (16.0)
Marital status	
With partner	215 (70.0)
Without partner	92 (30.0)
Religion	
Catholic	169 (55.0)
Non-Catholic	138 (45.0)
Hospital sector	
Ward	53 (17.3)
Intensive care unit	109 (35.5)
Emergency room	89 (29.0)
Other	56 (18.2)
Professional background	
Nursing technician	149 (48.5)
Nurse	124 (40.4)
Physician	23 (7.5)
Other	11 (3.6)
Post-graduate degree	
Does not have	200 (65.1)
Specialization	90 (29.3)
Master’s	15 (4.9)
Doctorate	2 (0.7)
Monthly income	
<USD 210	1 (0.3)
USD 211 to 630	136 (44.3)
USD 631 to 1050	116 (37.8)
>USD 1051	54 (17.6)
Variable	Mean (+/−SD)
Number of jobs	1 (1–2)
Weekly workload (hours)	12 (12–12)
Age (years)	35.53 (8.53)
Variable	Median (25th–75th percentile)
Time since graduation (months)	96 (36–156)
Work experience (months)	84 (24–144)
Time in sector (months)	24 (6–84)

**Table 2:** Analysis of factor loadings, communality, eigenvalues and variance explained by total and each factor of scale (*n* = 307).

Factor	Items	Factor 1	Factor 2	Factor 3	Communalities
Personal					
	1	0.889	0.195	0.126	0.827
	5	0.689	0.482	0.201	0.591
	8	0.705	−0.006	0.455	0.610
	9	0.838	−0.008	0.336	0.829
	14	0.706	0.038	−0.160	0.718
	18	0.878	0.185	0.260	0.836
	20	0.715	0.230	0.482	0.816
	24	0.794	0.198	0.038	0.716
	28	0.709	0.120	0.580	0.688
	37	0.863	0.154	0.199	0.805
	42	0.835	0.312	0.187	0.811
	45	0.847	−0.008	0.361	0.826
	47	0.776	0.315	0.459	0.723
Organizational					
	2	0.335	0.864	0.204	0.817
	7	0.068	0.755	0.442	0.660
	10	0.310	0.632	0.482	0.787
	13	0.199	0.875	0.121	0.825
	10	0.485	0.682	0.392	0.720
	12	0.211	0.808	0.141	0.717
	15	0.122	0.599	0.468	0.592
	17	0.133	0.653	0.354	0.589
	21	0.392	0.623	0.482	0.768
	23	0.415	0.710	−0.006	0.567
	25	0.258	0.768	0.494	0.815
	27	0.429	0.713	0.289	0.810
	29	0.200	0.833	0.164	0.801
	30	0.392	0.732	0.428	0.784
	31	0.415	0.789	0.233	0.763
	32	0.434	0.709	0.159	0.692
	34	0.317	0.658	0.464	0.751
	35	0.212	0.728	0.444	0.815
	36	0.378	0.632	0.261	0.593
	39	0.193	0.653	−0.085	0.602
	40	0.318	0.865	0.157	0.818
	43	0.104	0.729	0.442	0.693
	44	0.412	0.785	0.323	0.754
	46	0.336	0.684	0.482	0.786
Psychosocial					
	3	0.330	−0.007	0.847	0.826
	6	0.310	−0.159	0.798	0.784
	11	0.408	0.210	0.889	0.842
	16	0.134	−0.043	0.717	0.617
	19	0.205	−0.309	0.798	0.689
	22	0.187	0.465	0.720	0.697
	26	0.342	0.120	0.680	0.587
	33	0.004	0.591	0.166	0.377
	38	0.178	0.255	0.696	0.623
	41	0.482	0.380	0.632	0.786

Variance explained by factor: 13.28%, 51.52%, 7.5%; total variance explained: 78.2%.

**Table 3:** Spearman’s correlation coefficients (rho) between total score of the Adherence to Good Hospital Practices for COVID-19 and its domains (*n* = 307).

	ASPC	PERS	ORG	PSYCH
AGHPC	1			
PERS	0.841 *	1		
ORG	0.907 *	0.705 *	1	
PSYCH	0.665 *	0.485 *	0.416 *	1

AGHPC—Adherence to Good Hospital Practices for COVID-19; PSYC—psychosocial domain; ORG—organizational domain; PERS—personal domain; * correlation is significant at 0.01 level (two-tailed).

**Table 4:** Internal consistency (Cronbach’s alpha) for Adherence to Good Hospital Practices for COVID-19 and its domains (*n* = 307).

	N. of Items	Cronbach’s Alpha
AGHPC	47	0.96
PERS	13	0.88
ORG	24	0.95
PSYCH	10	0.61

AGHPC—Adherence to Good Hospital Practices for COVID-19; PSYCH—psychosocial domain; ORG—organizational domain; PERS—personal domain.

**Table 5:** Median (25th–75th percentile) of the temporal stability distribution for Adherence to Good Hospital Practices for COVID-19 and its domains (*n* = 31).

Test-Retest	M1 *	M2 **	ICC ***
AGHPC	74 (67.5–77.5)	183 (173–188)	0.742 (0.77–085)
PSYCH	15 (13–18)	38 (35.5–40.0)	0.710 (0.62–0.68)
ORG	38 (34–42)	95 (87.5–99.5)	0.874 (0.89–0.95)
PERS	17 (16–20)	49 (45.5–52.0)	0.768 (0.78–0.81)

AGHPC—Adherence to Good Hospital Practices for COVID-19; PSYCH—psychosocial domain; ORG—organizational domain; PERS—personal domain; * Moment 1—first interview; ** Moment 2—second interview after 7 to 14 days, *** ICC—intraclass correlation coefficient.

## Data Availability

The data that support the findings of this study are not publicly available because they contain potentially identifying or sensitive patient information that compromises the privacy of the research participants. However, an anonymous dataset can be made available from the corresponding author under reasonable request. Data requests should be sent to Silmara Meneguin, e-mail: s.meneguin@unesp.br.
